# Observations of specimen morphology effects on near-zone-axis convergent-beam electron diffraction patterns

**DOI:** 10.1107/S1600576724001614

**Published:** 2024-03-21

**Authors:** Xiaofen Tan, Laure Bourgeois, Philip N. H. Nakashima

**Affiliations:** a Department of Materials Science and Engineering, Monash University, Victoria 3800, Australia; b School of Materials Science and Engineering, Nanchang Hangkong University, Nanchang 330063, People’s Republic of China; c Monash Centre for Electron Microscopy, Monash University, Victoria 3800, Australia; Shiv Nadar Institution of Eminence, India

**Keywords:** convergent-beam electron diffraction, nano-structured materials, multislice theory, electron crystallography, specimen symmetry

## Abstract

This work presents observations of symmetry breakages in the intensity distributions of near-zone-axis convergent-beam electron diffraction (CBED) patterns that can only be explained by the symmetry of the specimen and not the symmetry of the unit cell describing the atomic structure of the material. The specimen is an aluminium–copper–tin alloy containing voids many tens of nanometres in size within continuous single crystals of the aluminium host matrix. Several CBED patterns where the incident beam enters and exits parallel void facets without the incident beam being perpendicular to these facets are examined. The symmetries in their intensity distributions are explained by the specimen morphology alone using a geometric argument based on the multislice theory. This work shows that it is possible to deduce nanoscale morphological information about the specimen in the direction of the electron beam – the elusive third dimension in transmission electron microscopy – from the inspection of CBED patterns.

## Abstract 2

Symmetries in the intensity distributions of convergent-beam electron diffraction (CBED) patterns are governed not only by the atomic structure of the unit cell but by the morphology of the diffracting specimen as a whole. A simple interpretation based on structural tilt projections and the multislice scattering theory may prove useful in extracting morphological information embedded in the presence or absence of CBED pattern symmetries for a wide range of applications, from 4D scanning transmission electron microscopy to bonding electron density studies in nano-structured materials.

## 1. Introduction

The intensity distributions in diffraction patterns from single crystals are dependent not only on the structure of the unit cell but also on the morphology of the specimen (Johnson, 1972 ▸; Goodman, 1975 ▸). Specimen shape and symmetry, while always accounted for in the accurate interpretation of diffracted intensities in any type of single-crystal diffraction experiment (Maslen & Spadaccini, 1993 ▸), are generally of minor interest in themselves because the primary focus in crystallography is usually the determination of the atomic structure.

Any atomic structure solution depends on the determination of structural symmetry within the unit cell (*i.e.* its space group), and convergent-beam electron diffraction (CBED) has long been established as the most sensitive method for space group determination, being able to determine, independently and unequivocally, 218 of the 230 space groups (Tanaka, 2010 ▸; Tanaka & Tsuda, 2011 ▸). The caveats are that CBED is only practical if the specimen can withstand high-energy focused electron beams with minimal damage incurred (new low-dose methods are proving powerful in extending the application of CBED), and if the unit cell is small enough to avoid the overlap of reflections that makes their intensity distributions difficult to interpret, though large-angle rocking-beam electron diffraction can eliminate this issue (Koch, 2011 ▸; Koch *et al.*, 2012 ▸). Fortunately, neither of these problems impede the present work.

The sensitivity of CBED to atomic structure is a consequence of the very strong dynamical scattering of electron beams from atomic potentials. Such scattering is many orders of magnitude stronger than X-ray scattering from the electron distribution around atoms or neutron scattering from atomic nuclei (Valvoda, 2006 ▸). This means that specimens suitable for CBED are at most a few hundred nanometres thick. Coupling this with the ability to focus electron beams to form probes smaller than a few nanometres results in CBED being able to sample volumes of material that are typically 10^9^ times smaller than the volumes probed by X-ray diffraction. Furthermore, the ability to position electron probes with sub-nanometre precision while imaging the region of interest in a transmission electron microscope (TEM) provides CBED with nanometre (or better) spatial selectivity.

The spatial selectivity of CBED has most frequently been exploited to obtain patterns from regions of perfect single crystals for space group determinations (Tanaka, 2010 ▸; Tanaka & Tsuda, 2011 ▸), bonding charge density studies (Zuo *et al.*, 1999 ▸; Nakashima *et al.*, 2011 ▸), specimen-thickness measurements (Spence & Zuo, 1992 ▸; Zuo & Spence, 2017 ▸) and TEM high-tension calibration (Fitz Gerald & Johnson, 1983 ▸), to name a few examples among many applications. However, on occasion, CBED has also been used to probe defects (Johnson, 1972 ▸; Zhu *et al.*, 2017 ▸). Here, we use the spatial selectivity of CBED to collect patterns through voids embedded in single-crystal host matrices of an aluminium–copper–tin alloy. We note that such a diffraction experiment would be impossible with any other diffraction technique and, therefore, the information that the present experiments yield is unique.

Particular attention is given to CBED patterns collected in orientations where the incident beam is not perpendicular to the parallel entrance and exit facets of the voids considered here. It is observed that the intensity distributions in reflections satisfying the Bragg condition do not possess the same symmetries as the patterns from an uninterrupted single crystal in the same incident orientations unless special geometric conditions are met concerning the location of voids with respect to the entrance and exit faces of the specimen. This is explained entirely by the specimen geometry and its structural projection along the incident beam direction – an argument that becomes self-evident when one considers the multislice scattering formalism (Cowley & Moodie, 1957 ▸). This will be substantiated in an experimental context with multislice-based quantitative CBED (QCBED) pattern matching (Nakashima, 2002 ▸; Streltsov *et al.*, 2003 ▸; Peng & Nakashima, 2017 ▸; Zhu *et al.*, 2017 ▸; Nakashima *et al.*, 2021 ▸) of the experimental patterns presented herein.

## 2. Experiments and methods

Voids many tens of nanometres in size were produced within single crystals of an aluminium–copper–tin alloy (Al–1.7 at.% Cu–0.01 at.% Sn) by the same thermal shock and subsequent heat treatment methods as described by Tan *et al.* (2021 ▸) and Bourgeois *et al.* (2010 ▸). The same recipes for preparing TEM specimens as described by those authors were also used for the present specimens. The heat treatments ensured that the voids that were produced were large enough to allow the incident electron beam to be oriented slightly off the zone axis perpendicular to the main void facets [see Tan *et al.* (2021 ▸) for the facetted cross-sectional shape of these voids], so that the Bragg conditions of reflections with short primary scattering vectors could be satisfied while maintaining the condition that all beams enter and exit through parallel void facets. This is essential because, while multislice models do not require structural periodicity along the direction of the incident beam, they do assume 2D periodicity in the planes (slices) perpendicular to the incident beam direction, making them unsuited (along with any other scattering formalism) to simulating CBED patterns from wedged specimens. Furthermore, ensuring that the electron beams transmit through parallel facets means that, when considering the intensity distributions within the resultant CBED patterns, a combination of effects need not be considered and any analysis (qualitative or quantitative) is simplified by only considering uniform matrix–void–matrix slab thicknesses.

All experimental data presented here were collected with 200 keV (nominal energy) electrons using an FEI Tecnai G2 F20 S-twin field emission gun TEM (FEGTEM) and a Gatan Ultrascan 1000 CCD camera. Probe sizes of 5 nm and less ensured that the extent of the electron beam was always much less than the size of the void facets it was transmitted through.

All multislice-based CBED pattern calculations and QCBED pattern-matching refinements of thicknesses were performed with the in-house *QCBEDMS* software, co-written by Nakashima and Zuo (Spence & Zuo, 1992 ▸; Zuo, 1993 ▸; Nakashima, 2002 ▸; Streltsov *et al.*, 2003 ▸; Peng & Nakashima, 2017 ▸; Zhu *et al.*, 2017 ▸; Nakashima *et al.*, 2021 ▸). Because the objective of QCBED in this work was only to determine thicknesses and because the CBED data were sub-optimal for rigorous QCBED treatment, the step of point-spread function correction (Nakashima & Johnson, 2003 ▸) was unnecessary. All CBED pattern simulations and QCBED refinements included phenomenological absorption at the levels reported by Bird & King (1990 ▸).

## 3. Results and discussion

**Figure 1:**
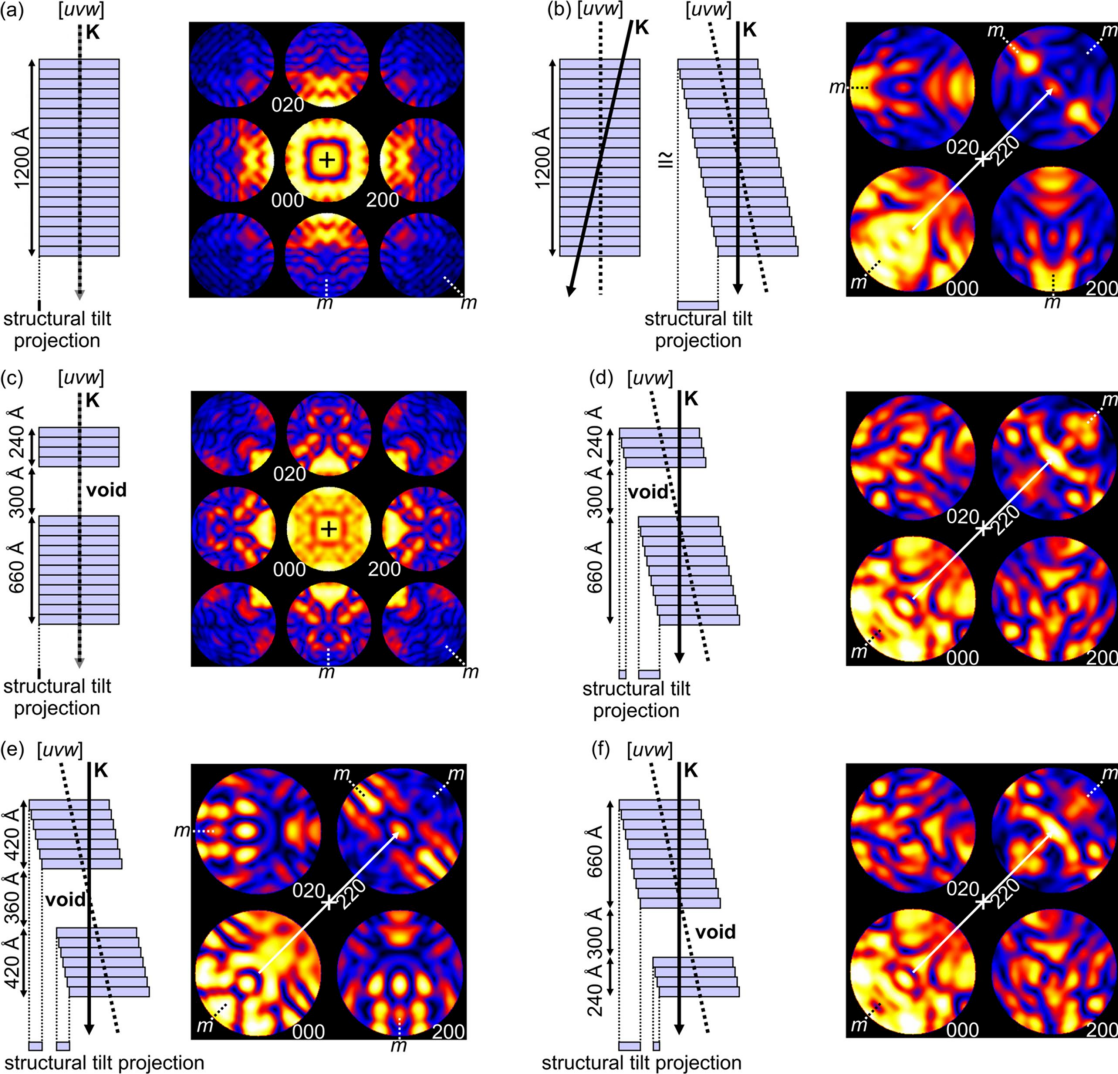
The effects of specimen morphology on diffracted intensities in CBED patterns from on-zone and tilted near-zone orientations. The present examples show multislice-simulated CBED patterns at or near the [001] zone axis in pure aluminium (*Fm* 3 *m* – a centrosymmetric space group) with 200 keV electrons. The total matrix–void–matrix thickness in all simulations shown here is 1200 Å, with individual segment thicknesses applied in the calculations of the patterns labelled in the accompanying schematic models. In cases (*a*) and (*c*) the cube root of the intensities and in cases (*b*) and (*d*)–(*f*) the square root of the intensities were taken to flatten the dynamic range and make the intensity distributions visible in all reflections. In all cases, the location of the zone-axis orientation is marked by a cross in the centre of the pattern. The schematic slicing models give the multislice representation of each situation, but with angles and slice dimensions exaggerated for illustrative purposes. The slice thicknesses shown in these diagrams correspond to 60 Å, while the slice thickness used in all multislice simulations was 2.0245 Å (*d*_002_ in aluminium). In all cases, the incident wavevector **K** is used schematically to indicate the centre of what in CBED would be a cone of incident wavevectors. (*a*) Zone-axis orientation where the incident wavevector **K** is perpendicular to the slices. (*b*) Tilting so that the incident beam is not parallel to the zone axis is equivalent to shearing the sequence of slices. (*c*)–(*f*) Removal of slices containing atomic structure allows voids to be simulated. (*c*) On-zone incident beam with a void closer to the entrance face of the specimen. (*d*) Tilted near-zone orientation in which the zone axis bis­ects the primary scattering vector (white) to the Bragg condition in the 220 reflection, where the void is closer to the entrance face of the specimen. (*e*) The same orientation as in (*d*) but with the void centred between the entrance and exit faces of the specimen. (*f*) The same orientation as in (*b*), (*d*) and (*e*) but with the void closer to the exit face of the specimen. The structural tilt projection given for every case shows the projection of periodically conjugate points in each slice in the incident beam direction. If the structural tilt projection is centrosymmetric, then the Bragg-satisfied reflection whose primary scattering vector is bis­ected by the zone axis will contain a centre of symmetry. This is not the case for (*d*) and (*f*).

This investigation begins by considering different geometric configurations of single crystals with and without voids, with the electron beam incident along the zone axis and in a tilted near-zone orientation, where the zone axis is the one perpendicular to the entrance and exit facets of the void. The different possibilities are illustrated in Fig. 1 ▸.

Only pure aluminium and [*uvw*] = [001] are considered for this part of the discussion and in all of Fig. 1 ▸. When the incident beam is parallel to this zone axis, the CBED patterns are expected to have 4*mm* symmetry (aluminium having a space group of *Fm* 3 *m*), as shown in Fig. 1 ▸(*a*) by the simulated CBED pattern, which, as with all others in Fig. 1 ▸, was calculated for 200 keV electrons. The location of the zone axis is marked by a black cross at the centre of the 000 reflection disc.

The schematic multislice model for this scenario in the case of an uninterrupted single crystal [left-hand side of Fig. 1 ▸(*a*)] shows the incident wavevector **K** parallel to the zone axis and perpendicular to the continuous slice sequence. In this and all other cases examined in Fig. 1 ▸, a cone of incident wavevectors (as is the case in CBED) has not been drawn and is replaced by a single incident wavevector for simplicity.

The ‘structural tilt projection’ is the projection of periodically conjugate points in each slice along the incident wavevector, and in the case of Fig. 1 ▸(*a*), where the incident beam is aligned with the zone axis, this projection has no extent at all. The structural tilt projection is thus centrosymmetric by default and therefore it is expected that there will be a reflection in the CBED pattern that contains a centre of symmetry in its intensity distribution. In this case of on-zone incidence, this is the 000 disc as its primary scattering vector, **g**_000_ (which has zero length) is bis­ected by the zone axis. Thus, in this trivial case, the centre of symmetry marks the location of the zone axis in the diffraction pattern.

At this point, it is worth stating the following axiom: whenever the projection of the diffracting structure in the direction of incidence is centrosymmetric, the reflection whose primary scattering vector (*i.e.* extending from 000 to the reflection) is bis­ected by the zone axis will contain a centre of symmetry in its intensity distribution (Pogany & Turner, 1968 ▸; Goodman & Lehmpfuhl, 1968 ▸; Buxton *et al.*, 1976 ▸; Tanaka, 2010 ▸; Tanaka & Tsuda, 2011 ▸). This axiom is central to all subsequent discussion.

On tilting the incident beam on the specimen (or equivalently the specimen with respect to the incident beam), one has a scenario akin to that illustrated in the first model drawn in Fig. 1 ▸(*b*). In the multislice theory, tilting off the zone defined by the slicing direction in the crystal structure can be accomplished via the propagator *or* by shearing the slices with respect to one another as illustrated in the second model of Fig. 1 ▸(*b*). In the limit of infinitesimal slice thickness, these alternative approaches of incorporating off-axis tilt become identical. However, to gain a qualitative understanding of the effects of specimen morphology on CBED pattern intensity distributions, slice shearing provides a far simpler avenue. This approach is adopted from this point forth.

Considering the second model in Fig. 1 ▸(*b*), if one projects periodically conjugate points in each slice along the incident wavevector, one obtains a structural tilt projection that is not infinitesimal but has an extent defined by the tilt angle (greatly exaggerated in Fig. 1 ▸ and typically ∼1° in reality) and the thickness of the specimen. In the present case of continuous aluminium, this projection is centrosymmetric along the tilt direction. The corresponding CBED pattern is then expected to contain a centre of symmetry in the intensity distribution of the reflection satisfying the Bragg condition and whose primary scattering vector is bis­ected by the zone axis. Indeed, in the simulated CBED pattern, 2*mm* symmetry is observed within the 220 reflection, with the inversion centre located at the end of the 220 scattering vector (from 000, in white), which is bis­ected by the zone axis (white cross). The presence of mirror symmetry expected about the Bragg conditions for the 200 and 020 reflections for the present space group (Pogany & Turner, 1968 ▸; Goodman & Lehmpfuhl, 1968 ▸; Buxton *et al.*, 1976 ▸; Tanaka, 2010 ▸; Tanaka & Tsuda, 2011 ▸) is also worth noting for this orientation.

If a void intercepts the incident beam which is parallel to the zone axis [Fig. 1 ▸(*c*)], regardless of the depth of the void in the specimen, a CBED pattern with 4*mm* symmetry and a centre of symmetry in the 000 disc can be expected as per the example in Fig. 1 ▸(*c*). This is because the structural tilt projection once again has no extent [as in the case of Fig. 1 ▸(*a*)] and is therefore centrosymmetric by default. In other words, the projection of the specimen symmetry (as opposed to the crystal symmetry) along the incident beam direction remains centrosymmetric irrespective of the presence and depth of a void, as long as the incident beam direction is parallel to the zone axis.

In Fig. 1 ▸(*d*), the case of a void that is closer to the entrance surface of the specimen, in an orientation where the zone axis bis­ects the 220 primary scattering vector, is presented. In this case, the structural tilt projection is no longer centrosymmetric and, therefore, the 220 reflection no longer possesses a centre of symmetry in its intensity distribution as shown in the corresponding simulated CBED pattern. Note that the mirror symmetries in both 200 and 020, previously observed in the scenario of Fig. 1 ▸(*b*), are also lost.

If, for the same orientation as in Figs. 1 ▸(*b*) and 1 ▸(*d*), the void was to be located equidistantly from the entrance and exit faces of the specimen as per Fig. 1 ▸(*e*), the structural tilt projection would be centrosymmetric and the 220 reflection would once again possess a centre of symmetry in its intensity distribution, located at the terminus of the primary 220 scattering vector which is bis­ected by the zone axis. The mirror symmetries in both 200 and 020, previously observed in the scenario of Fig. 1 ▸(*b*), are also present.

Finally, with the void located closer to the exit face of the specimen as in Fig. 1 ▸(*f*), both the structural tilt projection and the 220 reflection whose primary scattering vector is bis­ected by the zone axis are non-centrosymmetric. Once again, as in the case of Fig. 1 ▸(*d*), the mirror symmetries in both 200 and 020, previously observed in Figs. 1 ▸(*b*) and 1 ▸(*e*), are lost.

Most noteworthy is that none of the effects of symmetry breakage observed in Fig. 1 ▸(*d*) and 1 ▸(*f*) were due to changes in the atomic structure of the diffracting crystal. The maximum symmetry expected independently of the specimen mor­phol­ogy is of course determined by the symmetry of the underlying atomic structure; however, the absence of certain symmetry elements in some of the scenarios explored [Figs. 1 ▸(*d*) and 1 ▸(*f*)] can be attributed to the morphological symmetry of the specimen alone.

An important observation is that the intensities in the 220 reflections of Fig. 1 ▸(*d*) and 1 ▸(*f*) are reversed with respect to one another along the direction of specimen tilt, just like the structural tilt projections in these two scenarios are reversed with respect to one another along the tilt direction. This is a consequence of the void being located equidistantly from the entrance face of the specimen in Fig. 1 ▸(*d*) and from the exit face of the specimen in Fig. 1 ▸(*f*). Such a relationship could be useful when it comes to making morphological predictions based only on observing the intensity distributions within CBED patterns such as these.

**Figure 2:**
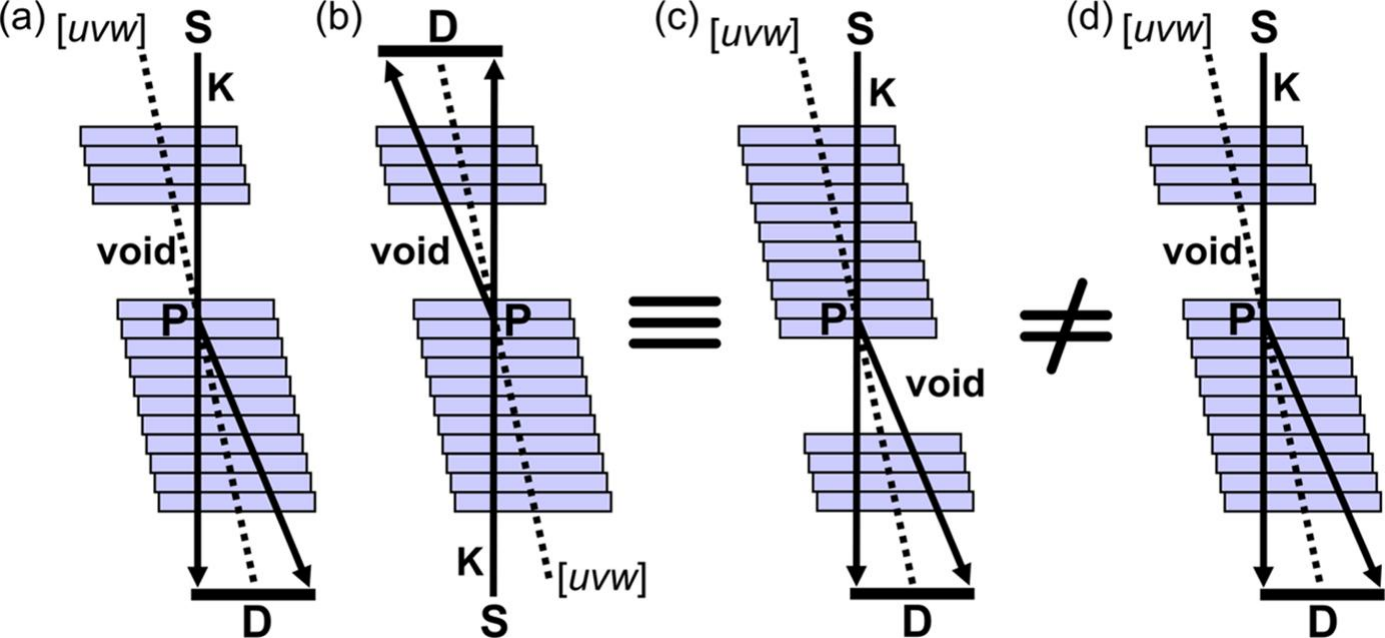
Alternative explanation for the intensity distribution differences in CBED patterns taken through voids of equal size that are equidistant in opposite directions from the specimen midplane, using the reciprocity theorem. (*a*) Repeat of the schematic illustrating the situation in Fig. 1 ▸(*d*). The electron source is labelled S, the detector is labelled D and one scattering event, labelled P, is considered, arbitrarily placed in the midplane of the specimen. (*b*) The source and detector have been exchanged with respect to P as per reciprocity. (*c*) Identical to (*b*), but rotated by 180°. (*d*) A copy of (*a*) to aid the comparison with (*c*). Note that the scattering event at P precedes the void in (*c*) but succeeds the void in (*a*) and (*d*) so the two scenarios are different, and one therefore expects different diffraction patterns as in Fig. 1 ▸(*d*) and 1(*f*).

Another approach to understanding why the intensity distributions in Figs. 1 ▸(*d*) and 1 ▸(*f*) are different is gained from the principle of reciprocity. This is illustrated in Fig. 2 ▸. Reciprocity dictates that if one exchanges the source and detector with respect to the scatterer, the same scattering amplitude will be recorded in both situations (von Laue, 1935 ▸, 1948 ▸; Pogany & Turner, 1968 ▸; Cowley, 1969 ▸). This depends on the scatterer being invariant under such a transformation. If one takes the example from Fig. 1 ▸(*d*) and introduces some scattering event, one arrives at the schematic in Fig. 2 ▸(*a*). Here the source is marked S, the detector is marked D and the scattering event, marked P, has been assigned to the midplane of the specimen. Exchanging the source and detector results in Fig. 2 ▸(*b*), which can be redrawn as in Fig. 2 ▸(*c*). This is a different scenario compared with Fig. 2 ▸(*a*), which is reproduced for comparison in Fig. 2 ▸(*d*), because in Fig. 2 ▸(*c*), the scattering event precedes propagation into the void whereas in Figs. 2 ▸(*a*) and 2 ▸(*d*) the scattering event succeeds propagation through the void.

**Figure 3:**
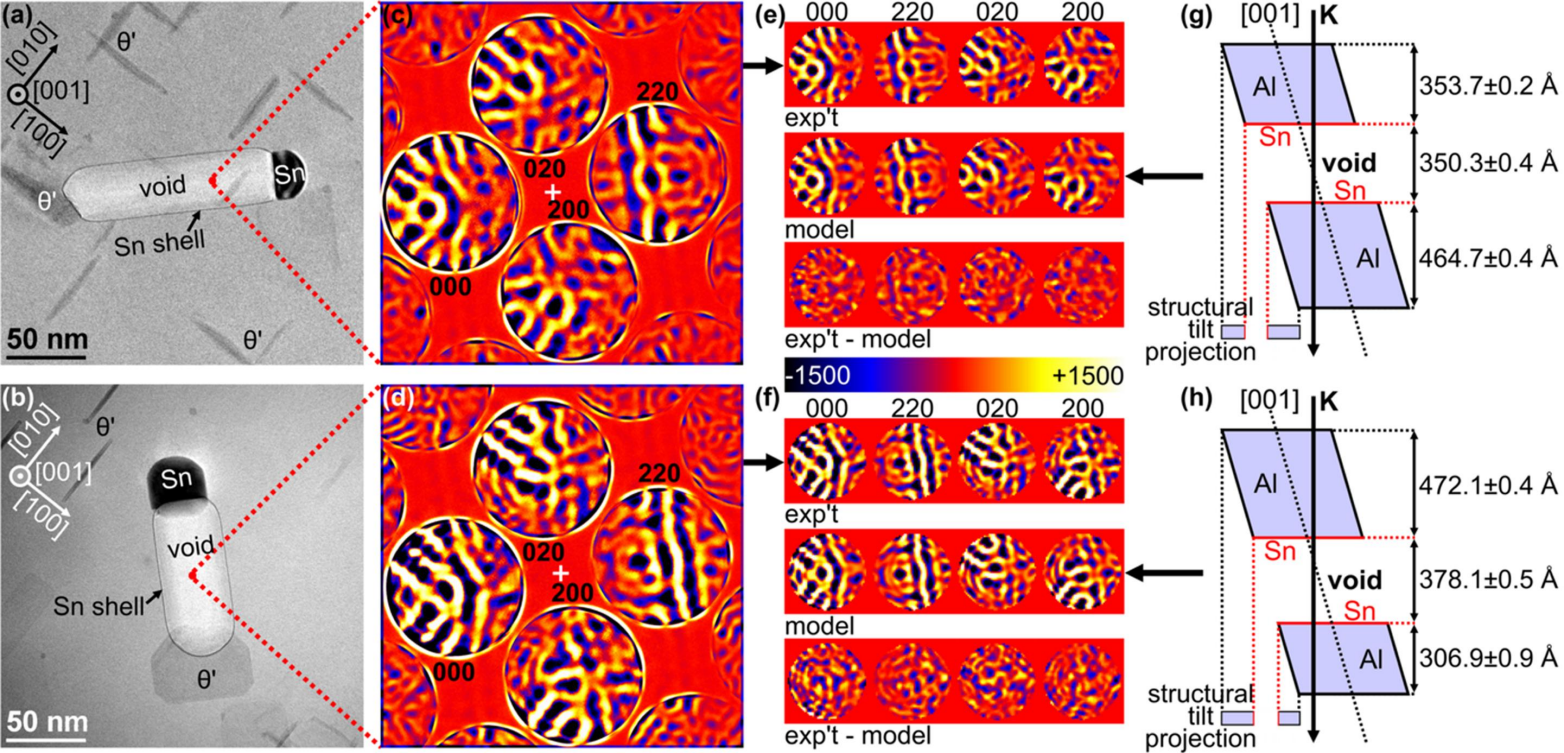
Experimental CBED patterns through two different voids in the same Al–Cu–Sn alloy specimen suggest that these two regions of the specimen have tilt projections that are related by inversion – implying that the voids in these two cases are equidistant from opposite specimen faces. (*a*) and (*b*) TEM images of the two different voids through which CBED patterns were collected at the indicated points (red dots). The voids, the associated monoatomic-thickness tin shell (Tan *et al.*, 2021 ▸), the connected tin precipitate, the connected θ′ and other independent θ′ precipitates are also indicated in these images. (*c*) and (*d*) CBED patterns from the indicated locations in (*a*) and (*b*), respectively, that have been radially differentiated (Nakashima & Muddle, 2010*a* ▸,*b* ▸) to remove the diffuse background due to inelastic scattering (a pre-requisite for QCBED) and to make turning points in the intensity distributions more distinct. (*e*) and (*f*) The differentiated CBED patterns from (*c*) and (*d*) furnished QCBED refinements of the thicknesses of each of the regions of the matrix–void–matrix multislice models shown in (*g*) and (*h*). (*g*) and (*h*) The outcomes of these thickness refinements; the models are drawn to scale except for the off-axis tilt, which has been exaggerated for illustration. The structural tilt projections are non-centrosymmetric in both cases and are approximately inverted with respect to one another along the tilt direction, as are the corresponding intensities in the 220 reflections of (*c*) and (*d*) along the 220 scattering vector. The QCBED-measured thicknesses shown in (*g*) and (*h*) also demonstrate the approximate inverse relationship between void depths within the specimen for these two cases. Note that the total thickness of the specimen for the first case [(*a*), (*c*), (*e*) and (*g*)] is 1169 ± 1 Å and for the second case [(*b*), (*d*), (*f*) and (*h*)] is 1157 ± 2 Å.

Some experimental results are now examined. Fig. 3 ▸ presents two different voids [Figs. 3 ▸(*a*) and 3 ▸(*b*)] within the same specimen and through which CBED patterns were collected in orientations where the primary 220 scattering vector was bis­ected by the [001] zone axis in both cases [Figs. 3 ▸(*c*) and 3 ▸(*d*)]. Here is a situation in which the intensities in the 220 discs in both patterns appear, at least qualitatively and approximately, reversed with respect to one another in the off-axis tilt direction (*i.e.* along the 220 scattering vector). Neither CBED pattern – both radially differentiated to remove the diffuse background due to inelastic scattering (Nakashima & Muddle, 2010*a* ▸,*b* ▸) and to accentuate the turning points in their intensity distributions – shows a centre of symmetry in the 220 reflection. This implies that, in both cases, the voids are not equidistant from the entrance and exit faces of the specimen. From the apparent intensity reversal in one 220 reflection with respect to the other [comparing Figs. 3 ▸(*c*) and 3 ▸(*d*) with one another] *and* the similarity in the angular frequencies of the intensity distributions in the two patterns, one can predict that the total specimen thickness is very similar in these two regions of the specimen and that the matrix–void–matrix thicknesses will be approximately reversed with respect to each other.

To test this prediction, QCBED pattern-matching refinements of the matrix–void–matrix thicknesses were conducted using the *QCBEDMS* program [Figs. 3 ▸(*e*) and 3 ▸(*f*)]. The monoatomic-thickness tin coating surrounding these voids (Tan *et al.*, 2021 ▸) and seen as a dark outline in the TEM images [Figs. 3 ▸(*a*) and 3 ▸(*b*)] was also accounted for, even though its influence on the intensity distributions in the CBED patterns collected is likely to be small. These tin layers had their thicknesses fixed to a single slice each (*i.e.* 2.0245 Å), which corresponds to the *d*_002_ interplanar spacing in the aluminium host matrix structure relevant to the [001] slicing direction in the present multislice models. Structure factors were not refined but fixed at values for the aluminium matrix measured in a previous accurate bonding electron density study of aluminium (Nakashima *et al.*, 2011 ▸). Given that the matrix regions will contain dissolved copper at concentrations estimated at <1 at.% due to the presence of numerous θ′ precipitates in the vicinity of the CBED pattern acquisitions [Figs. 3 ▸(*a*) and 3 ▸(*b*)], the approximations applied in the present refinements are unlikely to introduce large systematic errors into the refined thicknesses. Having said that, the fitted, calculated CBED patterns do have some non-random differences from the experimental patterns that are noticeable without the aid of the difference maps at the bottom of Figs. 3 ▸(*e*) and 3 ▸(*f*). These variations are probably due to strain caused in the surrounding matrix by the tin particles that are invariably attached to voids in these alloys, as well as the nearby θ′ precipitates (which have a much more minor effect as they are semi-coherent with the aluminium matrix). Nevertheless, the similarities between the experimental and calculated patterns lend confidence to the present thickness determinations.

The outcomes of the QCBED refinements of the matrix–void–matrix thicknesses are shown in Figs. 3 ▸(*g*) and 3 ▸(*h*). The specimen morphologies measured from each CBED pattern by QCBED are drawn to scale except for the tilt angle which has been exaggerated for illustrative purposes. The uncertainties in the thicknesses are certainly underestimated but are stated as shown because they represent the variability of the parameters within the region of the optimum ‘goodness of fit’ in the ten cycles of QCBED pattern matching and geometric distortion correction applied according to the method of Nakashima (2005 ▸).

These results [Figs. 3 ▸(*e*)–3 ▸(*h*)] confirm what was qualitatively predicted from observations of the CBED pattern intensity distributions and their relationship to one another in Figs. 3 ▸(*c*) and 3 ▸(*d*) for both voids. The QCBED results confirm the following:

(1) The total specimen thicknesses containing both voids are very similar: *H*_Total_ = 1169 ± 1 Å in the case of the first void [Figs. 3 ▸(*a*), 3 ▸(*c*), 3 ▸(*e*) and 3 ▸(*g*)] and *H*_Total_ = 1157 ± 2 Å in the case of the second void [Figs. 3 ▸(*b*), 3 ▸(*d*), 3 ▸(*f*) and 3 ▸(*h*)].

(2) The void depths are approximately reversed for one case with respect to the other. The first void [Fig. 3 ▸(*a*), 3 ▸(*c*), 3 ▸(*e*) and 3 ▸(*g*)] is almost the same distance from the exit face of the specimen as the second void [Fig. 3 ▸(*b*), 3 ▸(*d*), 3 ▸(*f*) and 3 ▸(*h*)] is from the entrance face of the specimen.

The scale models and thicknesses show the morphological reversal along the incident beam direction just as their corresponding structural tilt projections show (qualitatively) a reversal in the tilt direction and both CBED patterns show intensity reversal along the tilt direction (*i.e.* along the 220 scattering vector).

## 4. Conclusions

Though specimen morphology is routinely considered in the accurate interpretation of diffracted intensities, regardless of the type of radiation used, it is usually of less interest than the structure within the unit cell describing the crystal of interest (for obvious reasons).

The present investigation has demonstrated, theoretically and experimentally, the very strong effects that the symmetry of the specimen morphology can have on the intensities in CBED patterns. This could be exploited for morphological contrast imaging in 4D scanning transmission electron microscopy – a major area of development in microscopy, crystallography and diffraction physics (Ophus, 2019 ▸).

Observations of CBED intensity distributions alone cannot lead to quantitative information about specimen morphology. They can, however, permit the qualitative prediction of some aspects of the specimen morphology, though their predictive capacity is going to be severely limited if these observations are made in isolation. For example: if one only had CBED patterns from one of the voids in Fig. 3 ▸, there would no longer be a framework for comparing the different specimen morphologies. On the other hand, if CBED through a void in a titled near-zone orientation has a centre of symmetry in the intensity distribution of the reflection whose primary scattering vector is bis­ected by the zone axis, one can definitively say that the void is located equidistantly from the entrance and exit faces of the specimen [see, for example, Fig. 1 ▸(*e*)].

What the present work has done is shown that a multislice approach can easily be used to both rationalize (qualitatively) and quantify (by QCBED) the effect of specimen morphology on the absence (or presence) of symmetry elements within CBED patterns. Furthermore, the present work suggests that tilted near-zone orientations where symmetry breakages occur due to specimen morphology may enhance QCBED structure factor measurements in and around nanostructures if the nanostructures are extensive enough to make tilted near-zone orientations practicable for multislice-based QCBED (Nakashima *et al.*, 2021 ▸). Elevated sensitivity is anticipated under such circumstances for the following reasons:

(1) Tilted near-zone orientations make QCBED more sensitive to the structure factors of reflections at or near Bragg conditions than on-zone orientations (Streltsov *et al.*, 2003 ▸; Nakashima, 2017 ▸; Aryal *et al.*, 2021 ▸).

(2) Reduced symmetry in CBED patterns is synonymous with more complex intensity distributions and therefore more constrained QCBED pattern matching.

Future work exploiting these factors holds exciting potential when it comes to probing a range of matrix-hosted nanostructures. This includes precipitates on the condition that they have matching 2D structural periodicities with the host matrix at their interfaces (Nakashima *et al.*, 2021 ▸).
